# Black Soldier Fly Larvae Adapt to Different Food Substrates through Morphological and Functional Responses of the Midgut

**DOI:** 10.3390/ijms21144955

**Published:** 2020-07-13

**Authors:** Marco Bonelli, Daniele Bruno, Matteo Brilli, Novella Gianfranceschi, Ling Tian, Gianluca Tettamanti, Silvia Caccia, Morena Casartelli

**Affiliations:** 1 Department of Biosciences, University of Milano, 20133 Milano, Italy; marco.bonelli@unimi.it (M.B.); matteo.brilli@unimi.it (M.B.); novellagianfranceschi@yahoo.it (N.G.); 2 Department of Biotechnology and Life Sciences, University of Insubria, 21100 Varese, Italy; d.bruno1@uninsubria.it; 3 Pediatric Clinical Research Center “Romeo ed Enrica Invernizzi”, University of Milano, 20133 Milano, Italy; 4 Guangdong Provincial Key Laboratory of Agro-animal Genomics and Molecular Breeding/Guangdong Provincial Sericulture and Mulberry Engineering Research Center, College of Animal Science, South China Agricultural University, Guangzhou 510642, China; tianling@scau.edu.cn; 5 BAT Center—Interuniversity Center for Studies on Bioinspired Agro-Environmental Technology, University of Napoli Federico II, 80138 Napoli, Italy; 6 Department of Agricultural Sciences, University of Napoli Federico II, 80055 Portici (NA), Italy

**Keywords:** *Hermetia illucens*, insect midgut, post-ingestion regulation, diet composition, midgut transcriptome, waste management

## Abstract

Modulation of nutrient digestion and absorption is one of the post-ingestion mechanisms that guarantees the best exploitation of food resources, even when they are nutritionally poor or unbalanced, and plays a pivotal role in generalist feeders, which experience an extreme variability in diet composition. Among insects, the larvae of black soldier fly (BSF), *Hermetia illucens*, can grow on a wide range of feeding substrates with different nutrient content, suggesting that they can set in motion post-ingestion processes to match their nutritional requirements. In the present study we address this issue by investigating how the BSF larval midgut adapts to diets with different nutrient content. Two rearing substrates were compared: a nutritionally balanced diet for dipteran larvae and a nutritionally poor diet that mimics fruit and vegetable waste. Our data show that larval growth performance is only moderately affected by the nutritionally poor diet, while differences in the activity of digestive enzymes, midgut cell morphology, and accumulation of long-term storage molecules can be observed, indicating that diet-dependent adaptation processes in the midgut ensure the exploitation of poor substrates. Midgut transcriptome analysis of larvae reared on the two substrates showed that genes with important functions in digestion and absorption are differentially expressed, confirming the adaptability of this organ.

## 1. Introduction

In animals, the regulation of food intake and post-ingestion mechanisms are two important and strategic aspects to ensure optimal performance and healthy conditions [1]. With regard to nutritional demands, studies performed on insects and vertebrates have established that diet balancing and protein:carbohydrate ratio (P:C ratio) are two sides of the same coin [2,3,4]. Indeed, food intake is primarily regulated by the amount of these macronutrients, and thus the P:C ratio influences not only growth and development, but also body composition, reproduction, aging, gut microbiota, metabolic homeostasis, and immune functions [2,3,4,5]. The optimal dietary P:C ratio varies among species and it can also change within species, depending on the developmental stage and even sex as a result of different physiological needs [3,6,7].

The optimization of food intake is undoubtedly a prerequisite to match nutrient demand. Nevertheless, fitness maximization is ultimately and decisively guaranteed by the ability of animals to implement effective and targeted post-ingestion adjustments [1]. The presence and robustness of these mechanisms is of particular relevance in generalist feeders. Their responses, which include the modulation of nutrient digestion and absorption, the redirection of metabolism, and regulation of excretory system activity, have evolved not only to maximize food exploitation in order to meet nutritional requirements in the case of poor (i.e., with diluted or extremely diluted nutrients) or unbalanced diets, but also to compensate for the extreme variability of intake composition [1,7]. In this scenario, the gut plays a key homeostatic role, which strongly impacts on overall animal performance and fitness [1].

The black soldier fly (BSF), *Hermetia illucens* (Linnaeus, 1758) (Diptera: Stratiomyidae), represents a very attractive model for studying these aspects. Currently, BSF has a leading role in studies concerning the development of waste management strategies [8]. The interest in this saprophagous insect stems from multiple reasons. BSF larvae have a high feed conversion ratio [8] and exhibit an astonishing capacity to grow on a wide variety of organic materials and residues of disparate origin that are transformed into valuable biomass [6,9,10,11,12,13]. The high nutritional value of the larvae, and in particular the extent and the qualitative profile of their protein content, makes them exploitable as animal feed constituent, while their grease represents suitable raw material for biodiesel production [8,12,14]. In addition, BSF larvae have been used as a model for in vitro digestion studies [15] and described as a source of bioactive compounds with high biotechnological and medical potential such as chitin (and its derivative, chitosan) and antimicrobial peptides [16,17,18]. Finally, proteins obtained from BSF larvae have been recently used for the production of bioplastics [19]. While the striking bioconversion potential of BSF is well proven, knowledge on how rearing substrate composition and moisture, which in turn may condition intestinal microbiota, and rearing conditions (e.g., temperature and insect density) can affect the performance and body composition of this insect still needs to be improved [8,12,20,21]. In this respect, the literature on BSF clearly shows that carbohydrate and protein content in the rearing substrate significantly influences larval developmental time, larval and pupal weight, and their nutritional value, and thus the overall performance of the biotransformation process [8,12,20,21].

The present study aims to investigate whether and how BSF larvae are able to exploit nutritionally poor diets thanks to post-ingestion midgut responses. We explored this issue by performing a comparative analysis of the midgut morphology and physiology of larvae reared on two different diets, i.e., a nutritionally balanced diet for dipteran larvae and a diet that mimics fruit and vegetable waste composition. Our choice was motivated by the challenging nature of fruit and vegetable waste as raw material for BSF-mediated bioconversion processes [22,23]. Although nutritionally poor, it has been successfully used to rear *H. illucens* larvae [10,11,24] and strategies to reduce or reuse this valuable biomass are already underway [22,23,25]. In addition, fruit and vegetable waste is produced in large amounts by large-scale retail trade and wholesale markets, and it is allowed by the European Union as a rearing substrate for insects destined for fish feed (EU Regulation 2017/893) [26].

This study, which takes advantage of the knowledge obtained in our previous studies on the morphology and physiology of BSF larval midgut [24,27], evaluates the flexibility of this organ in response to changes in diet composition. Along with basic parameters of larval performance on different diets, we provide here a detailed morphofunctional characterization of the midgut responses. In addition, a complementary transcriptomic analysis of this organ does not simply support the structural and biochemical analyses performed herein, but also represents a well-stocked platform on which future work concerning the BSF larval midgut can be developed.

## 2. Results

### 2.1. Nutrient Content of the Rearing Substrates

Since this study aims to analyze the response of the BSF larval midgut to different nutrient availability, we preliminarily determined the nutrient composition of the two diets used to rear the larvae. Differences in the nutrient content of rearing substrates, especially carbohydrate and protein concentration and their ratio, can trigger insect behavioral and physiological adaptations [2,3,4]. Table 1 reports the composition of Standard Diet (SD) [28], a balanced diet conventionally used to rear BSF larvae, and Vegetable Mix Diet (VMD), a diet mimicking fruit and vegetable waste, expressed on “as fed” and “dry matter” bases. The former refers to the diet as fed to the larvae (taking into consideration the amount of moisture), the latter is computed on a moisture-free basis. Even though the P:C ratio of the two diets (calculated from the crude protein content and the available carbohydrates, i.e., starch, glucose, and fructose) was comparable, being 0.6 for SD and 0.4 for VMD, the content of these nutrients was quite different. In particular, considering the diet composition expressed on “as fed” basis, crude protein and carbohydrate supply was lower in VMD, and this diet was nutritionally poorer than SD due to the dilution of these macronutrients (Table 1). Also lipids, which have fundamental structural and functional roles, were 12-times more diluted in VMD (Table 1). Therefore, the two diets can be effectively used to compare the growth performance of BSF larvae and investigate if post-ingestion responses of the midgut are activated to compensate low nutrient supply of the feeding substrate.

Since insects, and BSF larvae in particular [18], are rich in minerals and their micronutrient profile depends on the dietary substrate [18], the content of specific mineral micronutrients in the two diets was evaluated, too (Table 2). A comparison between SD and VMD evidenced that the former was richer in minerals, especially iron (the concentration was 10- and 40-times higher when considering the diet composition expressed on “dry matter” or “as fed”, respectively) (Table 2).

### 2.2. Larval Growth Rate

**Figure 1:**
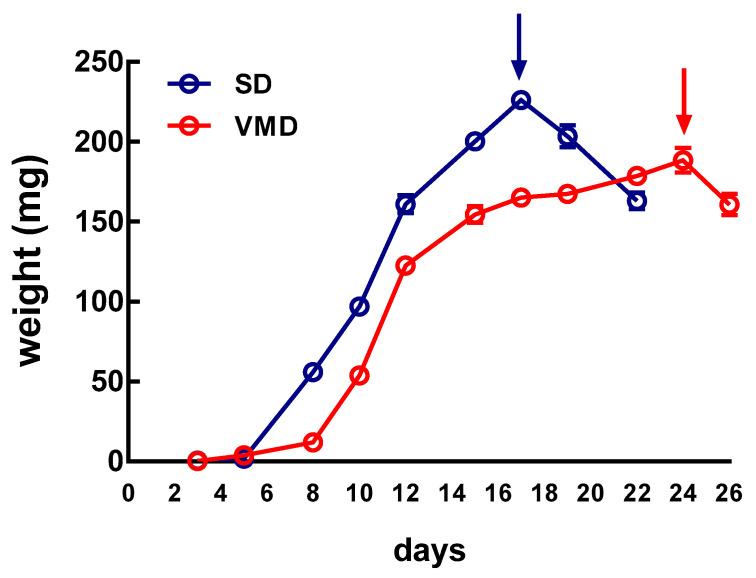
Growth rate of *H. illucens* larvae. The weight of the larvae reared on different substrates was recorded until 25% of insects reached pupal stage. The day in which the larvae reached the maximum weight was considered the end of the larval stage (arrows). Then insects entered the prepupal stage and stopped feeding.

The growth performance of BSF larvae reared on SD and VMD was evaluated. Figure 1 reports a typical experiment and shows that the duration of the larval stage, the end of which coincides with the attainment of the maximum weight before pupation (see “Measurement of larval growth rate” in Materials and Methods), was shorter when insects were grown on SD than on VMD (17 vs. 24 days, respectively). The maximum weight reached by the larvae was higher when larvae were reared on SD than on VMD (226.2 ± 3.7 mg vs. 188.5 ± 7.9 mg, respectively, mean ± s.e.m. of at least 20 samples, unpaired *t*-test, *p*-value < 0.001). These results are in agreement with our previous data [24] and show that larval growth performance, although affected, was not dramatically altered by the different nutritional content of the diets. Moreover, both larval groups were able to pupate and reached the adult stage.

### 2.3. pH of Diets and Midgut Lumen

The pH of freshly made SD and VMD was compared. The pH of SD was almost neutral, with a value of 6.8 ± 0.2 (mean ± s.e.m. of 5 samples), while the pH of VMD was acidic, with a value of 4.5 ± 0.2 (mean ± s.e.m. of 5 samples). Due to the different pH values of the diets, the possible effect of the feeding substrates on the pH of the midgut lumen was evaluated. This parameter has a critical role in midgut functionality and presents variations along the midgut of brachycerous Diptera, conferring peculiar functional features to each region of this organ [27,29]. pH was therefore measured in the anterior, middle, and posterior midgut of BSF larvae reared on SD and VMD. As reported in Table 3, no significant differences were found among the pH values recorded. These results are in agreement with pH values reported previously [27], being acidic in the lumen of the anterior midgut, strongly acidic in the middle, and alkaline in the posterior. Therefore, unlike previous reports on BSF larvae [30], the pH of the feeding substrate did not influence the midgut luminal pH, at least in our experimental conditions.

### 2.4. Enzymatic Assays

To assess if *H. illucens* larvae could modulate digestive activity in response to nutrient content of the feeding substrate, the activity of midgut enzymes involved in protein, carbohydrate, and lipid digestion was measured in larvae reared on both diets.

**Figure 2:**
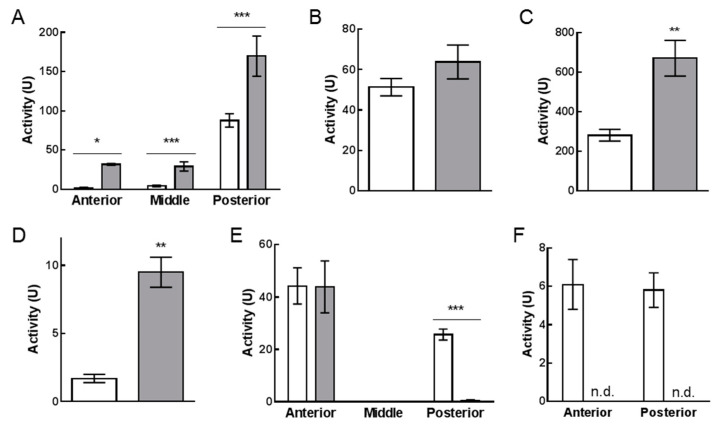
Enzymatic activities in midgut juice (**A**–**C**,**E**,**F**) or midgut homogenate (**D**) from larvae reared on SD (white bars) and VMD (grey bars). Total proteolytic activity in midgut juice extracted from anterior, middle, and posterior midgut (**A**). Trypsin- (**B**) and chymotrypsin- (**C**) like activity in midgut juice extracted from posterior midgut. Aminopeptidase N activity in the homogenate of posterior midgut (**D**). α-amylase activity in midgut juice extracted from anterior, middle, and posterior midgut (**E**). Lipase activity in midgut juice extracted from anterior and posterior midgut; n.d. non-detectable activity (**F**). The values are reported as mean ± s.e.m. of at least 3 experiments. Asterisks indicate statistically significant differences between diet groups (unpaired *t*-test: * *p*-value < 0.05, ** *p*-value < 0.01, *** *p*-value < 0.001).

As reported in Figure 2A, a significantly higher proteolytic activity was observed in all midgut regions of larvae reared on VMD, which has a lower protein concentration compared to SD (Table 1). The highest proteolytic activity was detected in the posterior region for both diets (Figure 2A), therefore, attention was focused on this tract for further analyses on protein digestion. Previous data on larvae reared on SD indicated that serine proteases, endopeptidases with an alkaline optimum pH, are the main enzymes involved in the initial phase of protein digestion in the posterior region of BSF larval midgut [27]. To evaluate if serine proteases were responsible for the increase of total proteolytic activity in this midgut tract in larvae reared on VMD, their activity was measured at acidic pH (pH = 5), which is very far from the pH optimum of these enzymes; in this condition a significant decrease of total proteolytic activity was observed (5-fold reduction, compared to pH = 8.5; total proteolytic activity at pH = 8.5: 169.7 ± 25.7 U, at pH = 5.0: 39.2 ± 7.2 U, mean ± s.e.m. of 4 experiments, paired *t*-test: *p*-value < 0.01). Given this evidence, the activity of trypsin and chymotrypsin, the two major serine proteases in insects [27,31,32], was measured in the posterior midgut. While no significant difference in trypsin-like activity among larvae reared on the two diets was observed (Figure 2B), chymotrypsin-like activity in larvae reared on VMD was double of that measured in larvae grown on SD (Figure 2C).

In addition to enzymes involved in the initial phase of protein digestion, enzymes responsible for the final phase of digestion were also considered. Among them, aminopeptidase N (APN), which is anchored to the midgut brush border membranes [31], was selected as representative of exopeptidase activity. APN activity in the posterior midgut was significantly higher in larvae reared on VMD, reaching values 6-fold higher than those measured in larvae grown on SD (Figure 2D).

To evaluate carbohydrate digestion, the activity of α-amylase was measured [27,31,32]. As reported in Figure 2E, no significant difference in α-amylase activity was observed in the anterior region and no activity was detected in both samples in the middle midgut, while larvae reared on VMD showed a significantly lower activity in the posterior region, with a 60-fold reduction compared to that measured in larvae reared on SD.

Finally, lipase activity was measured in anterior and posterior midgut. Middle midgut was not considered since lipase activity was not detectable in this region [27]. Significant activity was measured in both anterior and posterior midgut of larvae reared on SD (Figure 2F), in accordance with our previous data [27]. On the contrary, no lipase activity was recorded in both districts from larvae reared on VMD.

### 2.5. Morphological Analysis of the Larval Midgut

To evaluate if the feeding substrate could affect the morphology of midgut cells, a thorough microscopy analysis of the midgut districts from larvae grown on SD and VMD was done.

**Figure 3:**
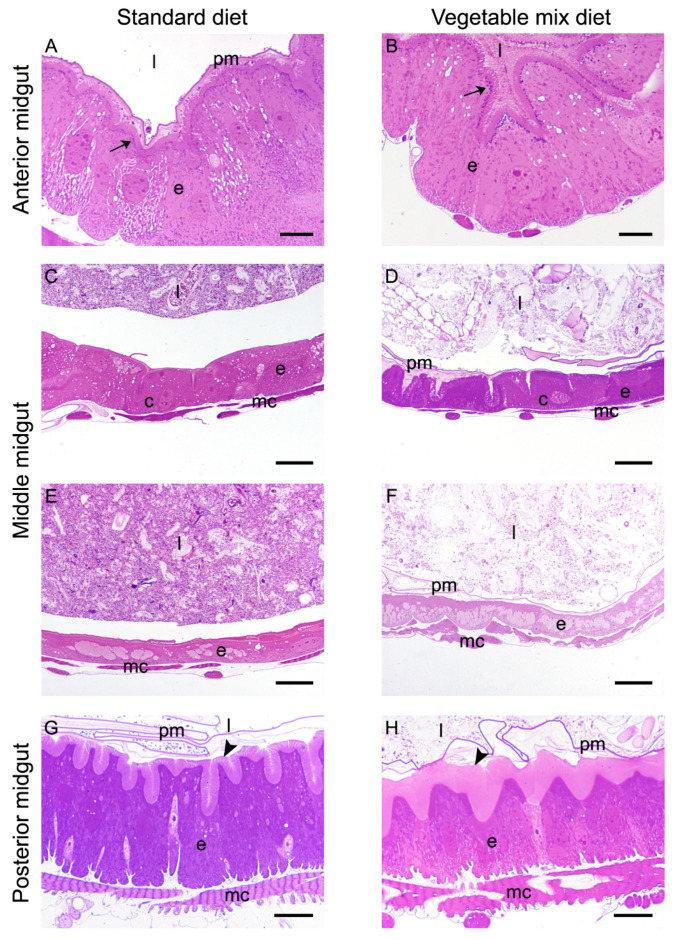
Morphological comparison of midgut from larvae reared on SD and VMD. (**A**,**B**): cross-sections of the anterior midgut. (**C**–**F**): copper (**C**,**D**) and large flat cells (**E**,**F**) in the middle midgut of *H. illucens* larvae. (**G**,**H**): cross-sections of the posterior midgut. Columnar cells of larvae grown on VMD (**H**) show microvilli (arrowheads) that are longer than those of columnar cells of larvae reared on SD (**G**). Arrows: dark vesicles under the brush border. c: copper cells; e: epithelium; l: lumen; mc: muscle cells; pm: peritrophic matrix. Bars: 10 μm (**A**,**B**), 20 μm (**C**–**H**).

The anterior midgut did not show any relevant modifications (Figure 3A,B): columnar cells of larvae reared on both substrates showed a big central nucleus, basal infoldings, and a well-developed brush border. Moreover, a large amount of dark vesicles, probably containing digestive enzymes, was clearly visible under the microvilli.

In the middle midgut of *H. illucens* larvae, the first tract formed by an epithelium containing copper cells is followed by a district in which a thinner epithelium presents large and flat cells [27,29]. Both cell types showed their typical morphological features regardless of the feeding substrate: copper cells exhibited the typical cup shape with a big central nucleus and developed microvilli (Figure 3C,D), while large flat cells presented an elongated nucleus and very short microvilli (Figure 3E,F).

The most consistent change in the morphology of the epithelium was observed in the posterior midgut (Figure 3G,H). In fact, the brush border of columnar cells showed a different length depending on the diet. In particular, columnar cells of larvae reared on VMD were characterized by microvilli that were longer than in larvae reared on SD, suggesting an increase in the absorbing surface.

### 2.6. Histochemical Characterization of the Larval Midgut

To get insights into the storage efficiency of the epithelium in relation to the diet, a histochemical approach was used. In particular, the storage of glycogen, which is important for energy production, was evaluated. Moreover, due to the importance of iron homeostasis in insects [33,34], the storage of iron was examined. It should be noted that iron metabolism initiates with its uptake from the diet by midgut cells, which also store iron by binding it to proteins [33,35].

**Figure 4:**
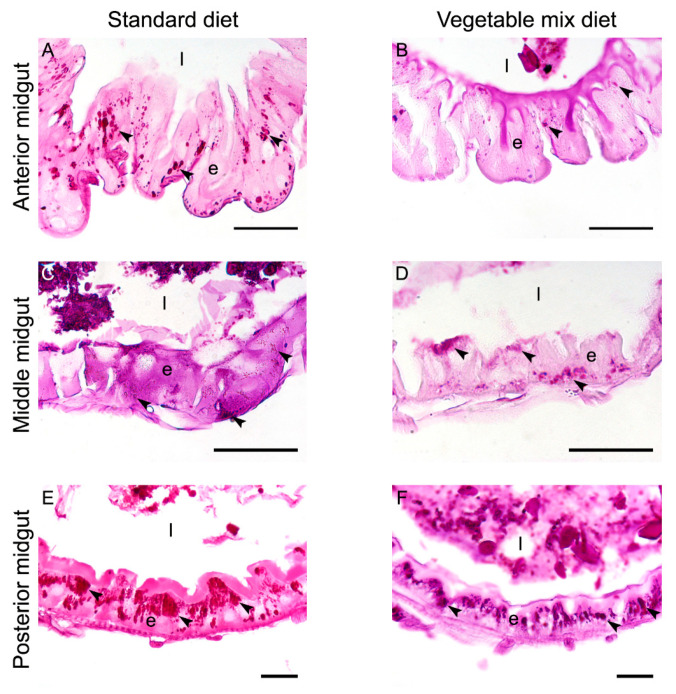
Comparison of glycogen accumulation in the three midgut regions of larvae reared on SD and VMD—Periodic Acid-Schiff (PAS) staining. (**A**,**B**): anterior midgut of larvae reared on SD (**A**) shows a higher accumulation of glycogen (arrowheads) than VMD (**B**). (**C**–**F**): middle (**C**,**D**) and posterior (**E**,**F**) midgut of larvae reared on the two diets do not show significant differences in glycogen accumulation. e: epithelium; l: lumen. Bars: 50 μm (**A**,**B**,**E**,**F**), 20 μm (**C**,**D**).

The comparison of glycogen deposits in the midgut of larvae reared on the two diets evidenced a major difference in the anterior midgut, where glycogen accumulation was more consistent in larvae reared on SD than on VMD (Figure 4A,B). Moreover, the deposits in this region were sparsely distributed throughout the cytoplasm rather than localized in a specific area (Figure 4A,B). In the middle and posterior midgut, glycogen deposits showed almost the same localization and abundance in larvae grown on both diets (Figure 4C–F), although the distribution differed in the two midgut regions. In detail, glycogen deposits were sparse into the cytoplasm in the middle midgut (Figure 4C,D), while in the posterior midgut they were localized in the apical region of columnar cells (Figure 4E,F).

**Figure 5:**
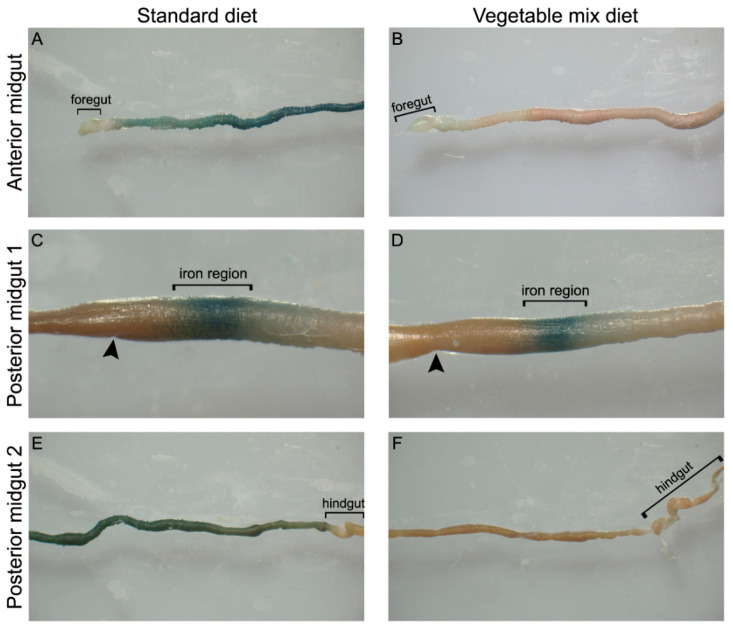
Comparison of iron accumulation between the three midgut regions of larvae reared on SD and VMD—Perls’ method. (**A**,**B**): a higher iron accumulation in the anterior midgut of larvae reared on SD (**A**) compared to VMD (**B**) can be observed. (**C**,**D**): iron region in the first part of the posterior midgut of larvae reared on the two diets. (**E**,**F**): higher accumulation of iron in the second part of the posterior midgut of larvae grown on SD than VMD is visible. Arrowheads: transition zone between middle and posterior midgut.

Since iron plays important physiological functions in insects [33,34] and our analyses showed that its concentration significantly differs in the two diets (Table 2), we examined the iron content in midgut cells. This mineral, which is particularly abundant in SD, showed a broader distribution in the midgut of larvae grown on this diet compared to those reared on VMD (Figure 5). The presence of this element in the first part of the posterior midgut in larvae reared on the two diets (Figure 5C,D) could be attributed to iron cells, a peculiar cell type that has been identified in the midgut epithelium of other Diptera [35]. Apart from this characteristic localization, in the anterior (Figure 5A,B) and in the second part of the posterior midgut (Figure 5E,F), the presence of iron in larvae reared on SD was higher than on VMD (Figure 5A,B,E,F). Regardless of the feeding substrate, the middle midgut did not show any staining (data not shown).

### 2.7. De Novo Transcriptome Analysis

RNA-Seq was performed on midgut samples isolated from *H. illucens* larvae reared on SD and VMD. Gene expression analysis was performed in triplicate for each diet (6 libraries in total), obtaining over 516 million reads (with an average of about 67 million reads per replicate). The reads were quality trimmed and the high-quality reads were normalized and used as input to perform de novo transcriptome assembly as reported in Materials and Methods (“De novo transcriptome and functional annotation”). Reads normalization reduced the redundancy of the dataset, allowing a less computationally intensive analysis and increasing the quality of the final assembly. This procedure resulted in a raw assembly of 32,643 transcripts with size ranging from 201 nt to 26,717 nt (average 1309 nt) and an N_50_ of 2392 nt. The average GC content was 41%. This preliminary assembly was filtered by using three different strategies (see “De novo transcriptome and functional annotation” in Materials and Methods for details). First, transcripts with very low expression level were removed, as they could be the outcome of erroneous reads and, moreover, they were extremely difficult to identify as differentially expressed, as the dispersion of the gene expression estimate is inversely correlated with the expression level. Second, very similar transcripts were merged with CD-HIT-EST to minimize the redundancy [36]. Third, all the transcripts with a best match to sequences from organisms outside the arthropods were removed, as midgut samples contain associated contaminating microorganisms. The features of the resulting transcriptome are reported in Table 4.

One important question mark in de novo transcriptome assembly is what fraction of the entire coding potential of the species of interest has been recovered, as it affects the understanding of processes under analysis. To assess the true coding potential, firstly the reads were mapped back to the assembly showing that 86.8% of them were included in the transcripts (86% map uniquely), and secondly, the transcriptome was compared against a dataset of genes that are universal in related genomes. For this purpose, BUSCO v3 [37], a tool that exploits a set of conserved single copy genes in a meaningful set of genomes to provide a measure of the completeness and redundancy of a genome/transcriptome assembly, was run. BUSCO pipeline was run with two different datasets: the first one contained a set of 303 proteins, which are conserved across Eukaryotes, while the second was more specific for our organism and comprised 1066 proteins that are conserved in all arthropod genomes. The output of the analysis showed that the present transcriptome was almost complete (Supplementary Table S1): it contained about 97% of the conserved single copy eukaryotic genes, 89% of which were estimated to be full-length and in single copy; consequently, only a small fraction of duplicated or fragmented genes was present. Similarly, our transcriptome contained about 94% of the Arthropoda conserved genes, 84% of which were complete and in single copy. The number of missing genes was about 3.5%. Taken together, these results show that the assembly can be considered a high-quality representative of the *H. illucens* midgut transcriptome.

**Figure 6:**
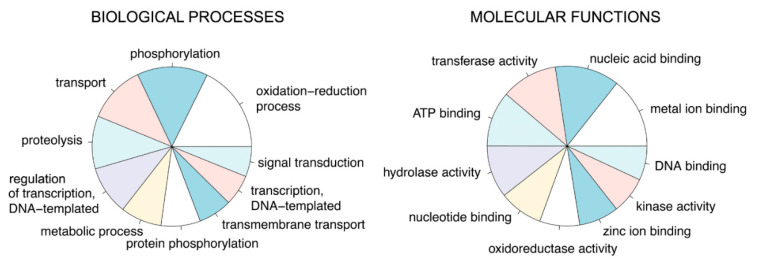
Graphical representation of the functional annotation of the transcriptome assembled in this work. Pie charts are realized using the CRAN platform and show the percentage of the 10 most represented gene ontology (GO) terms for Biological Processes and Molecular Functions. The categories are not terminal nodes in the GO hierarchy. The full list of categories is reported in Supplementary Table S3.

Once the quality and the degree of completeness of the de novo transcriptome of *H. illucens* midgut was verified, functional annotation of the transcripts was performed (Supplementary Table S2 in Supplementary file 1). To this aim, the Automatic assignment of Human Readable Descriptions pipeline (AHRD, [38]) was applied. The method is based on a similarity search between the transcripts and a reference set of proteins for which the annotation is known. Arthropoda proteins were used to assign a functional description to the transcripts, obtaining a gene ontology (GO) annotation for a total of 13,360 transcripts (Supplementary Table S3 in Supplementary file 1). The analysis of the annotation unveiled the abundance of transcripts related to “proteolysis” and “transport” in the Biological Processes (BP) category (Figure 6), in line with the prominent role of the midgut in digestion and transport of nutrients. Moreover, a high proportion of transcripts in BP category were involved in “phosphorylation” and “oxidation–reduction” processes (Figure 6). Within the Molecular Functions (MF) category, a bulk of transcripts pertained to “hydrolase activity” (Figure 6), confirming the importance of such function in *H. illucens* midgut. Other MF categories with a high number of transcripts were “metal ion binding”, “nucleic acid binding”, “transferase activity”, and “ATP binding”, suggestive of a metabolic active organ (Figure 6).

Two *H. illucens* genome assemblies are available at the NCBI genome assembly database [39]. The first, ASM101489v1 (GCA_001014895), has over 319K scaffolds and a N_50_ of only 1212 bp and was obtained in the framework of a work on transitions of sex chromosomes among Diptera [40]; the second assembly, indicated as “representative genome” for this species, is ASM983516v1 (GCA_009835165) and consists of 2806 scaffolds, and a N_50_ of over 1.69 Mbp [41]. By comparing our transcriptome assembly with the latter, a blast hit for 98.6% of our transcripts was found, 88.3% of which (i.e., 24,367 transcripts) sharing an identity level along the alignment of at least 90% together with a query coverage of 90% or more. The unavailability of gene or transcript sequences for the ASM983516v1 assembly did not allow further comparisons, but the above data confirmed the degree of completeness and general quality of our transcriptome.

### 2.8. Differential Gene Expression Analysis

**Figure 7:**
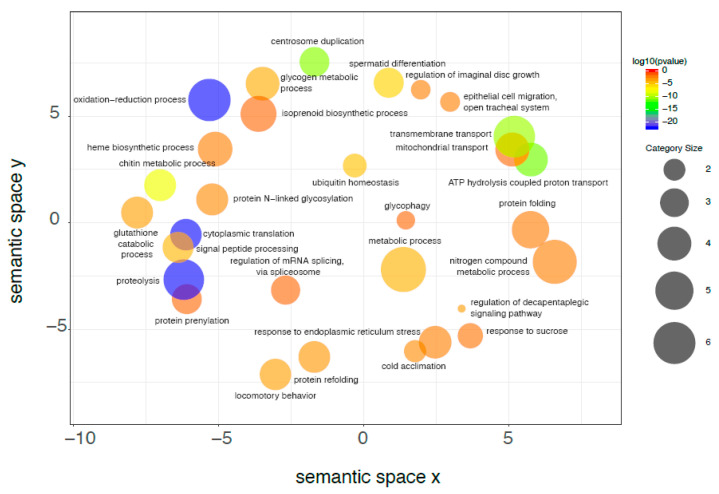
Upregulated genes associated to the GO category “Biological Processes”. Starting from the enrichment analysis of genes that are upregulated in midguts of larvae reared on VMD compared to SD, REVIGO was used to group similar biological processes on the basis of the SimRel semantic similarity metric; in this way, categories with similar descriptions are close in the plot. As shown in the scale on the right, the color of the bubble identifying each biological process is a function of log_10_ (*p* value) for the false discovery rate for the enrichment of each process. Bubble size indicates the frequency of each GO term (larger size indicates larger categories) and was calculated by REVIGO on the basis of the size of each category in a background database (SwissProt [42]). Only categories with FDR ≤ 1.0 × 10^−3^ were selected, see full list in Supplementary Tables S3–S7.

**Figure 8:**
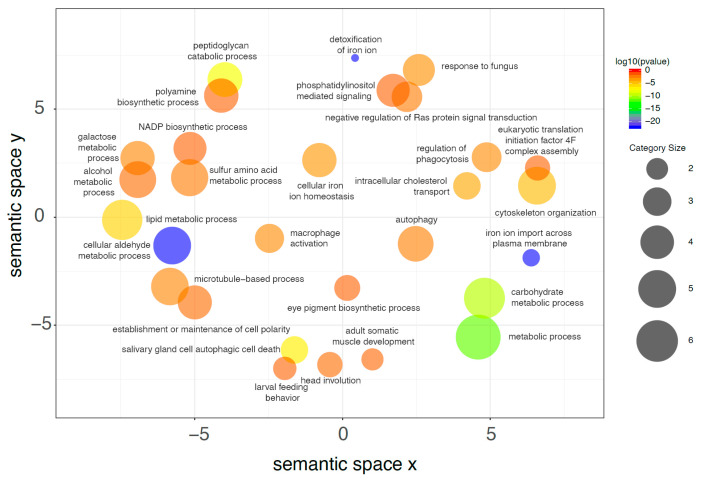
Downregulated genes associated to the GO category “Biological Processes”. Starting from the enrichment analysis of genes that are downregulated in midguts of larvae reared on VMD compared to SD, REVIGO was used to group similar biological processes on the basis of the SimRel semantic similarity metric; in this way categories with similar descriptions are close in the plot. As shown in the scale on the right, the color of the bubble identifying each biological process is a function of log_10_ (*p* value) for the false discovery rate for the enrichment of each process. Bubble size indicates the frequency of each GO term (larger size indicates larger categories) and was calculated by REVIGO on the basis of the size of each category in a background database (SwissProt [42]). Only categories with FDR ≤ 1.0 × 10^−3^ were selected, see full list in Supplementary Tables S3–S7.

GO annotation suggests the functional meaning of gene expression differences in midgut of *H. illucens* larvae reared on SD and VMD. The identification of differentially expressed genes provides a high-resolution view of the changes occurring in this organ as a consequence of the different rearing substrate, although it does not help understand large-scale functional modifications induced by the differentially expressed genes. For this reason, the examination of the transcriptomic changes in the midgut due to diets with different nutritional content was firstly performed by discussing the enrichment analysis of BP that exploits the GO annotation of the differentially expressed genes. This kind of approach provides a less detailed description of the transcriptomic changes (Figure 7; Figure 8; Supplementary Tables S3–S7 in Supplementary file 1), but it is highly informative because it shows the functional consequences associated with the multitude of transcripts that changed their expression level. Figure 7 shows that upregulated genes in VMD were significantly associated to GO categories related to proteolysis, translation, transport, and several metabolic processes (for instance “Glycogen metabolic process”). This evidence can be tentatively explained by the lower energy supply and protein content of the VMD that drives the midgut to optimize protein catabolism, increase nutrient uptake, and maintain high protein synthesis in support of the massive secretory activity (“Signal peptide processing” was also enriched). Conversely, the enrichment analysis of the genes significantly decreasing their expression level (Figure 8) highlights a reorganization of cytoskeleton and several metabolic processes, together with a high representation of categories related to iron. As VMD has a lower nutrient content, a reduced activity of some metabolic and catabolic processes is needed but sufficient to process the ingested nutrients.

The analysis of differentially expressed genes in the midgut of *H. illucens* larvae reared on SD and VMD identified 843 upregulated and 1067 downregulated transcripts, for a total of 1910. To unravel the functional adaptations of *H. illucens* midgut in response to the different rearing substrates, differential expression of genes coding for digestive enzymes and transport proteins was analyzed in detail.

**Figure 9:**
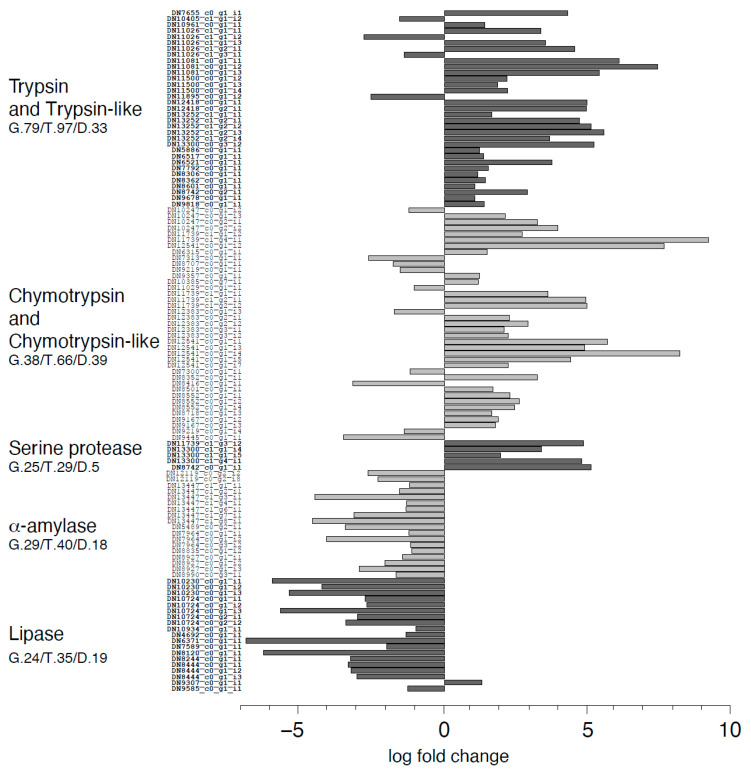
Log fold change of differentially expressed transcripts assigned to molecular functions corresponding to hydrolytic activity related to digestion in midguts of larvae reared on VMD compared to SD. For each function the number of genes (G.), transcripts (T.), and transcripts differentially expressed (D.) identified by the transcriptome analysis are shown together with their log fold change in our transcriptomic data.

**Figure 10:**
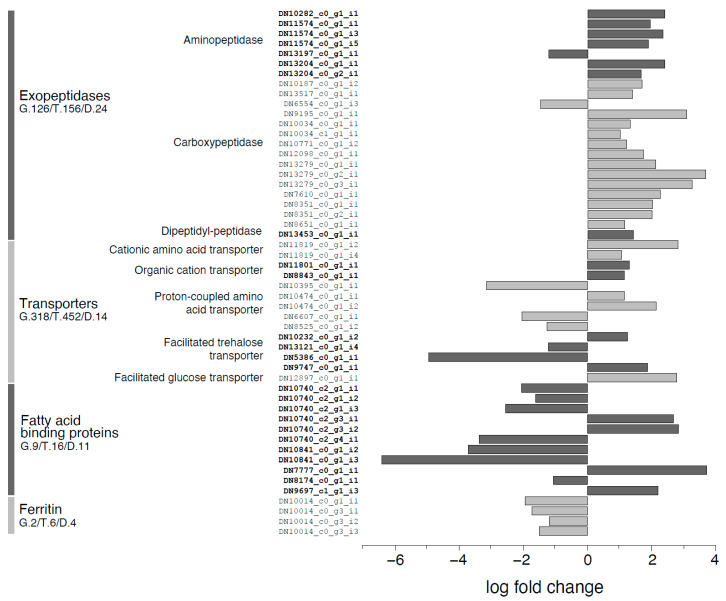
Log fold change of differentially expressed transcripts assigned to molecular functions corresponding to exopeptidases, membrane transport, lipid binding and transport, and iron binding in midguts of larvae reared on VMD compared to SD. For each function the number of genes (G.), transcripts (T.) and transcripts differentially expressed (D.) identified by the transcriptome analysis are shown together with their log fold change in our transcriptomic data.

Proteolytic enzymes were among transcripts increasing their expression in larvae reared on VMD (Figure 9). In particular, transcriptomic analysis showed that trypsin and trypsin-like transcripts were strongly enriched (29/97 transcripts), with fold change ranging from 2 to over 168, whereas only 4 out of 97 transcripts were downregulated. Similarly, 29/66 chymotrypsin and chymotrypsin-like transcripts were significantly upregulated for VDM (in a range of fold change from 2 to 570), whereas only 10/66 transcripts were downregulated. Although among serine proteases only 5 out of 29 transcripts were differentially expressed, all of them were strongly upregulated. It is noteworthy that exopeptidases were strongly upregulated in larvae reared on VMD (Figure 10). Conversely, the expression of transcripts coding for enzymes involved in carbohydrate and lipid digestion was downregulated in the midgut of larvae reared on VMD (Figure 9). The transcriptomic data showed that 18/40 transcripts coding for α-amylases were significantly downregulated with transcripts that were up to 20 times less abundant than in larvae reared on SD. Finally, lipases were strongly downregulated (Figure 9), too, with 19/35 transcripts differentially expressed, mostly downregulated (18/19).

A focus on transcripts involved in transport and binding of nutrients (Figure 10) revealed that only 14 transcripts out of 452 annotated as transporters were differentially expressed, and the expression changes were in both directions. A similar pattern was observed for lipocalins and fatty acid binding proteins, for which, however, 11/16 transcripts were differentially expressed. Finally, most of ferritin transcripts showed coordinated downregulation (4/6) in larvae reared on VMD.

## 3. Discussion

Insects are widely distributed in terrestrial ecosystems. They are characterized by an incredible variety of feeding habits thanks to an unmatched diversity of morphofunctional specializations and adaptability to cope with changes in diet composition [29]. The digestive apparatus, which is responsible for food ingestion and processing, represents a major player in this adaptation [29]. In this scenario, the study of the mechanisms that allow strict dietary specializations of insects is attractive, especially in the case of feeding substrates that are indigestible from a human perspective [43,44]. On the other hand, the mechanisms underlying the high efficiency of the digestive tract of highly polyphagous insects are worthy of investigation. These insects need to adapt to variations in nutrient composition of the ingested food, and *H. illucens* larvae, which are able to grow on a variety of organic waste materials and residues, are a relevant model to address this issue [6,9,10,11,12,13,25]. Moreover, the use of BSF in the feed sector has been promoted by recent changes in the EU legislation that partially lifted the feed ban rules regarding the use of processed animal proteins from insects (EU Regulation 2017/893) [26]. Thus, knowledge on the mechanisms that allow BSF larvae to exploit substrates with different nutritional quality is also relevant from an applied perspective.

In the present work we evaluated larval growth performance, as well as investigated the activity of digestive enzymes, morphological features of cells, changes of long-term storage molecules, and differential gene expression in the midgut of BSF larvae reared on two different diets, i.e., Standard Diet (SD) for dipteran larvae [28] and Vegetable Mix Diet (VMD). Whereas the former is a nutritionally balanced feeding substrate, the latter is nutritionally poor due to the lower concentration of protein, lipid, starch, and minerals compared to SD. These diets are thus particularly suitable to understand if and how the midgut sets in motion post-ingestion responses to compensate variations in nutrient composition of the diet because, although highly different from a nutritional point of view, they allow comparable larval growth performance, as demonstrated in the present study and in another recent work [24].

We initially focused attention on the digestion of proteins, in which the concentration in VMD is 5-fold less than in SD and could thus represent a limitation for larval performance. It has been demonstrated that BSF larvae strongly rely on serine proteases for protein digestion, which is mainly accomplished in the posterior midgut where most of tryptic and chymotryptic activities have been measured [27]. Enzymatic assays performed on larvae reared on the two diets showed an increase of total proteolytic activity in all midgut tracts of the larvae reared on VMD. These functional data were supported by an increase of transcripts coding for proteolytic enzymes (i.e., trypsin, trypsin-like, chymotrypsin, chymotrypsin-like, and other serine proteases). However, conversely to chymotrypsin and chymotrypsin-like enzymes, we did not observe any significant increase of proteolytic activity mediated by trypsin and trypsin-like enzymes. This could be due to the different extent of the regulation of genes coding for the two serine proteases. In fact, while chymotrypsin and chymotrypsin-like transcripts related to 27 of the 38 annotated genes were differentially expressed (mostly upregulated), a relatively limited number of genes were subjected to regulation for trypsin and trypsin-like enzymes (i.e., 24 out of 79). Thus, since for trypsin and trypsin-like proteins the enzymatic activity from constitutively expressed genes represents the bulk of the total activity, the impact of upregulated transcripts might be negligible. The overall increase of total proteolytic activity associated with enzymes involved in the initial phase of protein digestion was accompanied and corroborated by an increase of exopeptidases transcription (aminopeptidases and carboxypeptidases), which are involved in the final protein digestion [31]. Both functional (aminopeptidase activity) and transcriptomic data showed an increased ability of the midgut to obtain free amino acids from the diet. These are in turn internalized into midgut cells by apical transporters, the transcripts of which were also upregulated in larvae reared on VMD. This scenario suggests that, due to the low protein concentration in VMD, the larvae of *H. illucens* optimize the different steps of proteolysis and maximize the internalization of amino acids to meet their nitrogen requirements. Many studies in insects demonstrated that the expression and activity of serine proteases increase with the amount of proteins in the dietary substrate when these are not limiting nutrients [45,46], while complete lack of protein, as during starvation [47,48,49,50] or between blood meals in hematophagous insects [45,51,52], leads their activity to decrease or even drop. In our study, proteins, which are scarcely represented in VMD, might be a limiting nutrient, and therefore *H. illucens* larvae set in motion compensatory mechanisms to make the best use of this rearing substrate.

Our data also demonstrate a significant regulation of α-amylases in relation to diet composition. These enzymes catalyze the hydrolysis of α-1,4 glycosidic bonds in polysaccharides (essentially starch and glycogen) to produce oligosaccharides that are then hydrolyzed into glucose units by α-glucosidases. The regulation of midgut α-amylase activity by diet composition has been observed in several insects, although responses are quite variable even within the same order [53]. Indeed, in polyphagous lepidopteran larvae, midgut α-amylase activity responds to carbohydrate composition of the diet, but the nature of the correlation is inconsistent and discrepant results are reported [54,55,56,57]. For example, Sarate et al. [57] demonstrated that amylase activity in the midgut of *Helicoverpa armigera* (Lepidoptera: Noctuidae) is inversely proportional to carbohydrate content in the diet, in contrast to Kotkar et al. [55] that showed no direct correlation in this insect. On the other hand, gut amylase activity in *Spodoptera frugiperda* (Lepidoptera: Noctuidae) larvae increases with food consumption and carbohydrate amount [54,56]. In *H. illucens* larvae, α-amylase activity is mainly localized in the lumen of anterior and posterior midgut [27], whereas it is negligible in the middle midgut. The present study indicates that diet composition is able to strongly regulate α-amylase activity associated to the posterior midgut, while the activity is unaffected in the anterior midgut. In particular, the low starch concentration in VMD compared to SD was associated to the nearly complete absence of α-amylase activity in the posterior midgut. This trend was supported by the analysis of differential gene expression that showed an overall decrease of α-amylase transcripts in the whole midgut. The apparent discrepancy between the overall expression decline and the presence of unaltered α-amylase activity in anterior midguts may be due to the limited impact of transcripts associated to the relatively short anterior midgut compared to the posterior tract [27]. Alternatively, since about a half of the transcripts annotated as α-amylase were not differentially expressed, α-amylase genes could be constitutively expressed in the anterior midgut, while their expression could be regulated in the posterior midgut in response to carbohydrate content of the diets. Considering that in our transcriptome the number of genes and transcripts assigned to α-amylase are 29 and 40, respectively, and that the downregulated transcripts (18) in larvae reared on VDM originate from 14 genes, it is possible to conclude that the regulation of amylolytic activity involves a significant number of genes. Although in larvae reared on VMD starch hydrolysis occurs only in the anterior midgut, the activity of α-amylase in this tract and the amount of free sugars present in the diet appeared to be sufficient to meet larval requirements. Nevertheless, the larvae responded to low starch concentration in VMD with a slight increase of α-glucosidases, enzymes involved into the final phases of starch digestion (i.e., transcript DN11894_c0_g1_i2), and, a strong increase (7–fold) in the expression of sugar transporters to maximize the internalization of free sugars (i.e., transcript DN12897_c0_g1_i1). In accordance with these data, α-amylase activity in larvae of *Drosophila* spp. (Diptera: Drosophilidae) is increased by dietary starch and a similar trend was observed in other insect species, such as *Periplaneta americana* (Blattodea: Blattidae) and *Gryllus bimaculatus* (Orthoptera: Gryllidae) [58,59,60,61,62,63]. Interestingly, in the phytophagous insect *Locusta migratoria* (Orthoptera: Acrididae) α-amylase activity is regulated by the P:C ratio of the diet rather than by carbohydrate content [1]. In particular, when carbohydrate content of the diet is high, α-amylase activity is reduced only in the presence of low protein content. The occurrence in *H. illucens* larvae of glucose repression (i.e., the inhibition of α-amylase activity by the final products of starch digestion) observed in several *Drosophila* spp. [53,59] could not be determined in our experimental conditions (i.e., with fixed amounts of starch), but such regulation is worthy of further investigations.

Along with glycogen, lipids represent essential energy reservoirs and insects meet lipid requirements through de novo lipogenesis, which mainly occurs in the fat body, and dietary lipid digestion in the midgut lumen [64,65]. Lipases produce free fatty acids in the midgut lumen, that are then internalized into the midgut cells and converted into intracellular storage molecules or released as diacylglycerols into the hemolymph where they are shuttled by lipoproteins to other tissues [64]. VMD contains a 12-fold lower concentration of crude lipids than SD. *H. illucens* larvae grown on VDM did not increase the digestion of these nutrients in the lumen, but rather reduced the expression of lipases, which was accompanied by the drop of lipolytic activity. Accordingly, over half of the transcripts related to fatty acid binding proteins, that likely mediate the intracellular movement of absorbed fatty acids [64], were downregulated, too. The ability to regulate the expression and the activity of digestive lipases in response to the quantity and quality of dietary lipids has been detected in many insect species, and lipid deprivation, such as during starvation, is not always accompanied by lipolytic decrease [57,64,65,66,67,68]. In the case of *H. illucens* larvae reared on VMD, a basal, faintest lipase activity apparently guarantees the hydrolysis of available lipids. Indeed, although lower than in SD, the concentration of crude lipids is apparently sufficient to support larval growth.

Our results demonstrate that differences in the nutrient composition of the feeding substrate also induced modifications of midgut cells at a morphological level. Previous studies have shown that the diet can induce ultrastructural changes in insect midgut cells as fluctuations in the number and structure of lysosomes [69], proliferation of smooth and rough endoplasmic reticulum [70], and increase of the basal labyrinth surface [71]. Here, we observed a relevant change in the morphology of posterior midgut cells in larvae reared on VMD, which showed a significant increase in the length of microvilli. This modification, restricted to the region that is mainly involved in nutrient absorption [27], may respond to the need of a higher absorbing surface and likely represents an adaptation to the low nutritional content of VMD.

Histochemical analysis revealed differences in glycogen accumulation in *H. illucens* larval midgut. Glycogen reserves are crucial to sustain metabolic homeostasis throughout the life cycle and, in holometabolous insects, stored energy is useful during metamorphosis [72,73]. In *H. illucens*, this process occurs in about 12 days and glycogen deposits are mobilized and progressively reduced during this period [74]. Our data demonstrate that larvae reared on VMD show lower accumulation of this long-term storage molecule than larvae reared on SD, especially in the anterior midgut, suggesting that glycogen accumulation is reduced in favor of survival and growth when larvae experience a nutritionally poor diet. The higher presence of glycogen reserves in larvae reared on SD could be related to the higher content of nutrients in this diet that allows glycogen accumulation in the midgut. We observed that glycogen accumulation occurs differentially in the three midgut regions regardless of the feeding substrate. In particular, the anterior and the posterior midgut are mainly involved in glycogen storage, indicating that the cells in the midgut epithelium accomplish different metabolic functions [75]. Evidence suggesting the regionalization of glycogen and lipid metabolism in the midgut has already been obtained in adult *Drosophila melanogaster* [76,77]. Transcriptomic analysis was not particularly helpful in the interpretation of histochemical data.

Glycogen metabolism is mainly controlled by two enzymes, i.e., glycogen synthase and glycogen phosphorylase. From a survey of differentially expressed genes, it emerged that two transcripts were annotated as glycogen synthase (i.e., transcripts DN12700_c0_g1_i1 and DN12700_c0_g1_i2): the first was not differentially expressed, whereas the second was upregulated in larvae reared on VDM. Moreover, the unique transcript annotated as phosphoglucomutase (i.e., transcript DN12272_c0_g1_i) was not differentially expressed. Phosphoglucomutase controls the availability of glucose-1-phosphate, the precursor for glycogen synthesis, and is strongly downregulated in *D. melanogaster* mutants with reduced glycogen storage capacity [78]. On the other hand, although no transcripts annotated as glycogen phosphorylase were found, a number of transcripts that may correlate to a decrease in glycogen synthesis were downregulated in larvae reared on VDM. Indeed, 3 out of 4 serine/threonine kinase transcripts (i.e., enzymes that positively regulate glycogen synthase activity) were downregulated up to 4-fold, although the other one is upregulated. This not completely clear picture deriving from transcriptomic analysis may be due to the specific function of each midgut region, which, in turn, determines the different glycogen accumulation in the three districts.

Finally, we evaluated the accumulation of microelements in midgut cells of larvae grown on the two diets, focusing our attention on iron. Several studies demonstrated that this element is fundamental for the development of Diptera [79,80] due to its role as a cofactor for different enzymes involved in crucial physiological functions [81,82,83,84]. Ferritin is the major protein responsible for iron storage in insects [85] and is constitutively expressed in iron cells [86]. In *D. melanogaster* larvae these peculiar cells are located between the middle and posterior midgut, in a tract called “iron region” [87]. However, the expression of ferritin is inducible in other midgut cells in the fruit fly [86,88]. In fact, diets rich in iron induce the expression of ferritin encoding genes also in the anterior and posterior midgut cells, allowing iron accumulation in these regions [86,88]. In this study, we obtained comparable results. Cells able to accumulate iron are distributed in *H. illucens* larval midgut similarly to *D. melanogaster* and iron accumulation in these cells occurs regardless of the diet. By contrast, iron was accumulated also in the anterior and posterior midgut region only in larvae reared on SD, which is characterized by a higher iron content compared to VMD. In agreement with these data, ferritin encoding transcripts (4 out of 6) are downregulated in larvae reared on VMD.

## 4. Materials and Methods

### 4.1. Insect Rearing

*H. illucens* larvae used in this work were derived from a colony established in 2015 at the University of Insubria (Varese, Italy).

Two specific substrates, with different nutrient composition, were used to rear the larvae: (i) Standard Diet (SD) for dipteran larvae [28], composed of 50% wheat bran, 30% corn meal, and 20% alfalfa meal, mixed in the ratio 1:1 dry matter:water; (ii) Vegetable Mix Diet (VMD), composed by fruits and vegetables (apple, banana, pear, broccoli, zucchini, potato, and carrot) mixed in equal quantity and appropriately minced (i.e., cut into small pieces of about 5 mm). VMD mimics fruit and vegetable waste, and ingredients available throughout the year were chosen in order to standardize the experimental conditions. The rearing methods of the larvae on both diets were previously described [27,89]. Larvae were maintained at 27.0 ± 0.5 °C, 70 ± 5% relative humidity, in the dark. For all the experiments, last instar larvae were used.

### 4.2. Determination of Nutrient Content of the Diets

The analyses were conducted at the Department of Agronomy, Food, Natural Resources, Animals and Environment, University of Padua (Agripolis, Legnaro, Italy).

Three samples of fresh VMD were lyophilized with a freeze-dryer (Alpha 2-4 LD plus, Martin Christ GmbH, Osterode, Germany) under 12–15 mbar at –80 °C, and then analyzed to determine nutrient content. Samples of SD powder were analyzed as they were. Diet samples were analyzed for crude protein, crude lipid, crude fiber, nitrogen-free extract, and ash following the protocols of AOAC International [90,91]. Hemicellulose, cellulose, and lignin content was calculated from the concentration of neutral detergent fiber, acid detergent fiber, and acid detergent lignin determined as reported elsewhere [92] and following the protocols of AOAC International [90,91]. Starch was determined by enzymatic digestion followed by glucose quantification with HPLC. Free glucose and fructose were also quantified by HPLC.

### 4.3. Measurement of Larval Growth Rate

Batches of 300 larvae were grown in plastic containers. Starting from the fourth day after hatching, 20 individuals were randomly sampled every two days, washed in lukewarm tap water to remove rearing substrate debris from the body, wiped dry, and weighed. The weight was recorded until 25% of insects reached pupal stage. The day in which the larvae reached the maximum weight was considered the end of the larval stage, subsequently the insect entered the prepupal stage and stopped feeding (definition and description of the developmental stages of *H. illucens* are reported elsewhere [74]).

### 4.4. pH of Diet and Midgut Lumen

To evaluate pH of SD and VMD, 5 samples (1 g each) of fresh diets were placed in a plastic tube with 200 μL of distilled water. After mixing and short spinning, the liquid fraction was withdrawn and pH was measured by pH indicator strips with a resolution of 0.5 pH unit (Hydrion Brillant pH Dip Stiks, Sigma-Aldrich, Milano, Italy).

pH of the midgut juice (obtained as described below, section “Isolation of midgut samples”) from anterior, middle, and posterior region of last instar larvae reared on the two diets was measured using the same pH indicator strips. The experiment was repeated on six independent samples obtained from larvae reared on SD and VMD.

### 4.5. Isolation of Midgut Samples

After anaesthetization on ice with CO_2_, larvae were dissected and the midgut was isolated in Phosphate Buffer Saline (PBS) (137 mM NaCl, 2.7 mM KCl, 8.1 mM Na_2_HPO_4_, 1.76 mM KH_2_PO_4_, pH = 7.4) at 4 °C.

For measurements of midgut lumen pH, enzymatic assays, morphological analyses, and Periodic Acid-Schiff (PAS) staining, anterior, middle, and posterior midgut were collected, as previously reported [27]. In particular, for pH measurement and enzymatic assays (except for aminopeptidase N activity assay), the peritrophic matrix from different midgut regions, with the enclosed intestinal content, was isolated, centrifuged at 15,000× *g* for 10 min at 4 °C to remove the insoluble material, and supernatant (midgut juice) was collected. The midgut juice from 15 larvae was used as a fresh sample for the luminal pH measurements or stored at –80 °C for the enzymatic assays. The epithelium of the posterior midgut, devoid of the peritrophic matrix and collected from 15 larvae, was placed into cryovials and stored in liquid nitrogen for aminopeptidase N activity assay. For morphological analyses and PAS staining, the three midgut regions were processed as described below (“Optical microscopy analysis of the midgut epithelium” and “Histochemical analysis of the larval midgut”). For Perls’ staining and transcriptome analysis, the whole midgut epithelium, with the enclosed midgut content, was isolated and processed as described below (“Histochemical analysis of the larval midgut” and “RNA isolation and Illumina sequencing”).

### 4.6. Enzymatic Assays

Total proteolytic activity in midgut juice samples from anterior, middle, and posterior region, was assayed with azocasein (Sigma-Aldrich, Milano, Italy), measuring its degradation by release of azo chromophore [93], as previously reported [27]. For each midgut tract, the enzymatic assay was performed at pH as close as possible to that of the lumen (i.e., pH = 6.0 for the anterior midgut, pH = 5.0 for the middle midgut and pH = 8.5 for posterior). One unit (U) of total proteolytic activity with azocasein was defined as the amount of enzyme that causes an increase in absorbance by 0.1 unit per min per mg of proteins.

Chymotrypsin- and trypsin-like proteolytic activity in midgut juice samples were assayed with *N*-succinyl-Ala-Ala-Pro-Phe *p*-nitroanilide (SAAPPpNA, Sigma-Aldrich, Milano, Italy) and *N*α-Benzoyl-d,l-arginine *p*-nitroanilide hydrochloride (BApNA, Sigma-Aldrich, Milano, Italy), respectively, measuring their degradation by release of *p*-nitroaniline (pNA) [27]. These assays were performed at pH = 8.5 on midgut juice obtained from the posterior region. One unit (U) of chymotrypsin- and trypsin-like proteolytic activity was defined as the amount of enzyme that causes an increase in absorbance by 0.1 unit per min per mg of proteins.

The activity of APN was assayed using l-leucine *p*-nitroanilide as substrate, measuring its degradation by release of pNA, as previously reported [27]. Assays were performed on the posterior midgut epithelium that, after thawing, was homogenized in 50 mM Tris-HCl, pH = 7.5 (1 mL/mg tissue). One unit (U) of APN activity was defined as the amount of enzyme that releases 1 μmol of pNA per min per mg of proteins.

α-amylase activity in midgut juice samples obtained from anterior, middle, and posterior midgut, was assayed with starch as substrate, measuring its hydrolysis by the amount of maltose released, as previously reported [27]. The assay was performed at pH = 6.9. One unit (U) of α-amylase activity was defined as the amount of enzyme necessary to produce 1 mg of maltose per min per mg of proteins.

Lipase activity in midgut juice samples obtained from anterior and posterior midgut was assayed using a Lipase Activity Colorimetric Assay Kit (BioVision, Milpitas, CA, USA) according to the manufacturer’s instructions.

### 4.7. Statistical Analyses for pH Values, Larval Growth Parameters, and Enzymatic Activities

Statistical analyses were performed with R statistical software (ver. 3.6.1) [94]. Paired and unpaired *t*-tests were done. Statistical differences between groups were considered significant at *p*-value ≤ 0.05. The statistical analysis performed for each experiment and the *p*-values are reported in the captions to figures.

### 4.8. Optical Microscopy Analysis of the Midgut Epithelium

The three regions of the larval midgut, with the enclosed intestinal content, were processed for morphological analysis as previously described [27]. Briefly, after fixation in glutaraldehyde 4% (*v*/*v*) in 0.1 M Na-cacodylate buffer, pH = 7.4, specimens were dehydrated in an increasing ethanol series and then embedded in epoxy resin (Epon/Araldite 812 mixture). Sections of 0.6-µm-thickness were obtained with a Leica Reichert Ultracut S (Leica, Wetzlar, Germany), stained with crystal violet and basic fuchsin, and then observed under Eclipse Ni-U microscope (Nikon, Tokyo, Japan) equipped with TrueChrome II S digital camera (Tucsen photonics, Fuzhou, China).

### 4.9. Histochemical Analysis of the Larval Midgut

For glycogen detection, after dissection of the larva, the three regions of the midgut, with the enclosed intestinal content, were immediately fixed in 4% (*w*/*v*) paraformaldehyde in PBS for 2 h at room temperature and then overnight at 4 °C. After dehydration in increasing ethanol series, specimens were embedded in paraffin [72] and 7-µm-thick sections were obtained using a Jung Multicut 2045 microtome (Leica, Wetzlar, Germany). After deparaffinization, sections were stained with PAS kit (Bio-Optica, Milano, Italy), according to the manufacturer’s instructions, to detect the glycogen deposits in the midgut tissues, and then analyzed under Eclipse Ni-U microscope (Nikon, Tokyo, Japan) equipped with digital camera (Tucsen photonics, Fuzhou, China).

For the detection of ferric iron, whole mount staining of the entire midgut was performed. After isolation, the tissue was fixed in 4% (*w*/*v*) paraformaldehyde in PBS for 20 min, and then stained with Perls’ staining kit (Bio-Optica, Milano, Italy) according to the manufacturer’s instructions. Each region of the midgut was analyzed under NSZ-606 Zoom Stereo Microscope (Xiamen Phio Scientific Instruments, Xiamen, China) equipped with TrueChrome II S digital camera (Tucsen photonics, Fuzhou, China).

### 4.10. RNA Isolation and Illumina Sequencing

Larvae were reared on SD and VMD (3 replicates for each diet), anesthetized on ice, and washed in 70% ethanol (*v*/*v* in water) before dissection under sterile conditions. Midguts were isolated in autoclaved PBS in a sterile Petri dish (5.5 × 1.3 cm). For both rearing substrates, pools of 10 midguts for each of the 3 experimental replicates (a total of 6 samples) were collected in a cryovial containing TRIzol reagent (Thermo Fisher Scientific, Waltham, MA, USA) and kept at –80 °C until extraction of total RNA, which was performed according to the manufacturer’s instructions. Total RNA preparations were then treated with TURBO DNase I (Thermo Fisher Scientific, Waltham, MA, USA), according to the manufacturer’s instructions. Next Generation Sequencing (NGS) experiments, including samples quality control, were performed by Genomix4life S.R.L. (Salerno, Italy). RNA concentration in each sample was assayed with NanoDrop 1000 spectrophotometer (Thermo Fisher Scientific, Waltham, MA, USA) and its quality assessed with Agilent TapeStation 4200 (Agilent Technologies, Santa Clara, CA, USA). Indexed libraries were prepared from 1 µg of purified RNA from each sample with TruSeq Stranded mRNA Sample Prep Kit (Illumina, San Diego, CA, USA) according to the manufacturer’s instructions. Libraries were quantified using Agilent TapeStation 4200 (Agilent Technologies, Santa Clara, CA, USA) and pooled such that each index-tagged sample was present in equimolar amounts, with a 2 nM final concentration of the pooled samples. The pooled samples were subjected to cluster generation and sequencing using an Illumina Nextseq 500 (Illumina, San Diego, CA, USA) in a 2 × 100 paired-end format at 1.8 pmol final concentration.

### 4.11. De novo Transcriptome and Functional Annotation

The raw sequence files (fastq files), generated as reported above for each of the six samples, underwent quality control analysis using FastQC [95]. Quality check on the raw sequencing data allowed to remove low quality sequences while preserving the high-quality part of NGS reads. Filtering was performed with BBDuk [96] by specifying a minimum length of 35 nucleotides (nt), and a sequencing quality of at least 35. Supplementary Table S8 reports sequencing output for the six samples before and after quality filtering.

The high-quality reads were normalized to reduce redundancy and then assembled using Trinity v2.1.1 [97]. The raw transcriptome was then filtered as follows: (i) reads were mapped back on the transcriptome and transcripts with no expression, as determined using Kallisto [98], were removed; (ii) redundant sequences were merged using CD-HIT-EST [36]; and (iii) all the transcripts having a match to non-Arthropoda sequences were filtered out.

The quality of the assembly was estimated by (i) mapping the reads back to the assembly and (ii) using the BUSCO v3 pipeline [37] to get an estimate of the degree of completeness of the transcriptome assembly.

In order to obtain the expression quantification of the assembled transcripts in the 6 samples, Kallisto and the trimmed reads were used; abundance for all transcripts was expressed as TPM (number of transcripts per million); all the analyses in this study were based on this transcript abundance unit, after normalization using the trimmed mean of M-values formula [99]. The AHRD pipeline [38] was used to infer the putative function of the assembled transcripts. This method is based on a similarity search between the transcripts and a set of SwissProt databases [42] (Supplementary Table S2 in Supplementary file 1). For the analysis, the Arthropoda proteins were used; 48% of the input proteins were classified as “Unknown Protein”. The functional enrichment of the different samples was calculated using GO annotation of transcripts [100] and the hypergeometric cumulative distribution. The enrichment *p*-values were adjusted for multiple testing using the Benjamini–Hochberg correction (Supplementary Table S2 in Supplementary file 1).

### 4.12. Differential Gene Expression Analysis

The identification of the differentially expressed genes was performed with the package NOISeq [101], the threshold for significance used was FDR (false discovery rate) ≤ 0.05. A total of 4096 differential transcripts were found, of which 1671 were upregulated and 2425 downregulated (Supplementary Tables S3–S7 in Supplementary file 1). The subset of transcripts with at least a |log2(fold change)| ≥ 1 was considered (i.e., at least a 2-fold change, up or down, was considered of interest when statistically significant).

### 4.13. Availability of Data

The raw sequences are available at [102] with the study accession number ERP122672.

## 5. Conclusions

The present work definitely demonstrates that the midgut of *H. illucens* larvae is able to adapt to diets with different nutrient content and gives an important contribution to the ability of these insects to grow on a variety of feeding substrates. As a consequence of nutritionally poor diet, modifications of the digestive enzymatic machinery can be observed: (i) an increase in proteolytic activity and in the abundance of transcripts associated to serine-proteases, and (ii) a decrease in α-amylase and lipase activity and in abundance of related transcripts. Moreover, an increase in the length of microvilli of midgut cell ensures a higher absorbing surface and likely represents an adaptation to diet with a low nutritional content. Finally, a reduction of glycogen accumulation occurs. The overall picture suggests that midgut functions are regulated to modulate nutrient digestion and absorption with the aim of optimizing larval growth.

The study of the biochemical and molecular mechanisms underlying midgut plasticity, at the midgut level (e.g., role of peptides secreted by midgut endocrine cells) and/or involving other tissues (e.g., fat body, and nervous system) will be pivotal to complete the scenario here unveiled. Moreover, since an alteration of midgut microbiota was observed in larvae reared on the two substrates used in this study [24], the contribution of microorganisms in midgut digestive processes and thus in larvae adaptation to different substrates also represents a key issue to be investigated.

## Figures and Tables

**Table 1:** Chemical composition and moisture content of the two experimental diets. Values are expressed as g per 100 g of diet.

	Standard Diet		Vegetable Mix Diet	
Component	As Fed	Dry Matter	As Fed	Dry Matter
Crude protein	6.4	14.1	1.2	10.3
Crude lipid	1.2	2.7	0.1	0.7
Crude fiber ^a^	4.9	10.8	0.5	4.4
Nitrogen-free extract ^b^	30.2	67.3	9.6	80.0
Ash	2.3	5.1	0.6	4.6
Hemicellulose ^c^	9.8	21.3	0.4	3.6
Cellulose ^c^	4.4	9.7	0.6	4.6
Lignin ^c^	1.7	3.7	0.2	1.3
Starch	8.5	18.8	1.4	11.6
Glucose and fructose	1.5	3.3	1.5	12.8
Moisture	55	-	88	-

^a^ Includes most of cellulose and insoluble lignin. ^b^ Includes sugars, organic acids, pectins, soluble lignin, hemicellulose and a small percentage of cellulose. ^c^ Values calculated from neutral and acid detergent fiber analyses.

**Table 2:** Heavy metal content of the two experimental diets. Values are expressed as mg per Kg of diet.

	Standard Diet		Vegetable Mix Diet	
Component	As Fed	Dry Matter	As Fed	Dry Matter
Iron	119.1	261.8	3.1	26.9
Copper	4.2	9.3	0.8	6.8
Nickel	0.8	1.7	0.1	0.7
Zinc	22.4	49.3	2.1	18.1
Moisture	55	-	88	-

**Table 3:** pH values in the lumen of *H. illucens* midgut regions. Mean ± s.e.m., number of replicates in parenthesis. No statistically significant differences were recorded among diet groups for each midgut region (unpaired *t*-test).

	SD	VMD
Anterior midgut	5.8 ± 0.1 (6)	6.0 ± 0.1 (6)
Middle midgut	2.4 ± 0.2 (6)	1.8 ± 0.2 (7)
Posterior midgut	8.3 ± 0.4 (6)	8.8 ± 0.1 (7)

**Table 4:** Overview of the de novo transcriptome assembly of *H. illucens* midgut.

Sequencing and Assembly Parameters	Value
Total number of transcripts ^a^	27,102
Minimum length (nt)	201
Maximum length (nt)	26,717
Average length (nt)	1170
N_50_ of transcripts (nt) ^b^	2137
Reads remapped (%)	86.81

^a^ Number of transcripts resulting after the assembly by Trinity and the subsequent collapsing step by CD-HIT-EST, and after filtering out non-Arthropoda sequences. ^b^ N_50_ value represents the threshold delimiting 50% of the transcripts in the entire assembly which are equal to or larger than the reported value.

## Supplementary Materials

Supplementary Materials can be found at https://www.mdpi.com/1422-0067/21/14/4955/s1. Supplementary file 1 includes: Table S2. Functional annotation of the transcriptome by using SwissProt. Table S3. Functional annotation of the transcriptome using GO. Table S4. Expression levels of all transcripts in transcripts per million (TPM). Table S5. Differential expression analysis. Table S6. GO Enrichment analysis for downregulated transcripts. Table S7. GO Enrichment analysis for upregulated transcripts.

## Abbreviations

AHRD	automatic assignment of Human Readable Descriptions pipeline
APN	aminopeptidase N
BApNA	*N*α-Benzoyl-d,l-arginine *p*-nitroanilide hydrochloride
BP	biological processes
BSF	black soldier fly
P:C ratio	protein:carbohydrate ratio
FDR	false discovery rate
GO	gene onthology
MF	molecular functions
NGS	next generation sequencing
PAS	periodic acid-Schiff
PBS	phosphate buffer saline
pNA	*p*-nitroaniline
SAAPPpNA	*N*-succinyl-Ala–Ala-Pro-Phe *p*-nitroanilide
SD	standard diet
TPM	number of transcripts per million
VMD	vegetable mix diet
U	unit of enzymatic activity
