# Supplementary material for: Black Soldier Fly Larvae Adapt to Different Food Substrates through Morphological and Functional Responses of the Midgut
Source: Int J Mol Sci. 2020 Jul 13;21(14):4955. doi: 10.3390/ijms21144955 (PMC7404193; doi:10.3390/ijms21144955)
Supplement: Supplementary file 1 [file ijms-21-04955-s001.zip › Supplementary Tables - ijms-858388.docx]

**Supplementary Table S1.** Degree of completeness of the transcriptome assembly, evaluated using BUSCO. N indicates the number of conserved proteins.

| **Reference Dataset** | **Complete genes** | | **Fragmented genes** | **Missing genes** |
| --- | --- | --- | --- | --- |
|  | Genes in single copy | Duplicated genes |  |  |
| Eukaryotes (N=303) | 0.89 | 0.09 | 0.02 | 0.01 |
| Arthropoda (N=1,066) | 0.84 | 0.10 | 0.02 | 0.03 |

**Supplementary Table S8.** Sequencing output for the six samples before and after quality filtering.

| **Sample name** | **Treatment** | **N raw reads** | **N trimmed reads** |
| --- | --- | --- | --- |
| *H. illucens* SD1 | Larvae reared on standard diet, first rearing | 98,707,548 | 85,422,726 |
| *H. illucens* SD2 | Larvae reared on standard diet, second rearing | 97,735,100 | 84,485,002 |
| *H. illucens* SD3 | Larvae reared on standard diet, third rearing | 92,723,968 | 80,272,532 |
| *H. illucens* VMD1 | Larvae reared on vegetable mix diet, first rearing | 93,524,148 | 80,667,474 |
| *H. illucens* VMD2 | Larvae reared on vegetable mix diet, second rearing | 67,516,812 | 58,193,938 |
| *H. illucens* VMD3 | Larvae reared on vegetable mix diet, third rearing | 66,341,996 | 57,334,258 |
